# The Structure and Functions of the Female Breast, as They Relate to Its Health, Derangement, and Disease

**Published:** 1846-07

**Authors:** 


### ART. XIV.

*The Structure and Functions of the Female Breast, as they relate to its Health, Derangement, and Disease.* By E. W. TUSON, F.R.S., Surgeon to the Middlesex Hospital.—*London,* 1846. 8vo, pp. 485.

THE earliest impression produced by this book is, that—

" . . . . . . . one Mr. *Tuson*
Has taken up arms against English and Grammar;"

and it is an impression no less enduring (scarcely a page fails to give it fresh distinctness) than it is early. The preface introduces sundry examples of the belligerent propensities of Mr. Tuson against the parts of speech; and to illustrate these, as well as to give the reader some inkling of the aims and qualifications of this anti-grammatical warrior, we shall examine the contents of the said preface more closely than is our wont.

The book was, we learn, "compiled during those hours which could be spared from professional engagements, both in public and private practice,"—an intimation which might be regarded merely as a satisfactory indication of the marvellous extent of Mr. Tuson's practice, did it not serve to introduce a very remarkable apology for all the deficiencies of the subsequent pages. The practice caused interruptions, "which on some occasions broke the chain of reasoning already commenced, and perhaps may have prevented the same line of argument being continued; so that should the reader find any defect in this respect, he must attribute it to *this cause.*" Certainly, to this cause we shall invariably ascribe the contradictions, the total absence of reasoning power, nay, the want of common sequence of ideas, which throw a certain veil of obscurity over what would otherwise doubtless prove a mass of living light. Mr. Tuson's faculties were money-gathering ; people, too, would not let him alone,—and each absurdity he has put to paper is but an indirect proof of his social and intellectual greatness. And yet with touching modesty Mr. Tuson apologises for trespassing on public indulgence with this his seventh book. Let him take heart,—when individuals of his mental caliber condescend to produce books, what right have poor devils, like ourselves, to be nice? *They* may, it is true, be "prevented from continuing the same line of argument;" but what is *our* business, but to establish the missing nexus, and exult that we are permitted to exercise our intellects upon matter so profound? And Mr. Tuson talks so humbly of his early productions—his waistcoat-pocket Remembrancers, &c.—that our very hearts yearn towards a man so modest and yet so great. True, we do remember to have heard it said, there was a wondrous adaptation of the bulk of the said productions to the capacity of the intellect producing them, but for our parts we always saw in such observations naught but envy and malice. And does not the event justify us? Here is a book "as big as the best of them;" nor does its character allow the reader for a moment to imagine that Mr. Tuson has given the world one more illustration of the immortal fable of the Frog and the Bull.

Mr. Tuson's opportunities of observing have, he tells us, been great; but he feels "little satisfaction with the progress made in such a number of years." He has, however, "of late been *more* than particularly active [he was always, as is well known, particularly active] in his researches, and feels a *greater* gratification in having accomplished much more in a shorter space of time." Of course; all Mr. Tuson has to do at any time is to take to a subject in earnest, and he must do wonders; in the present instance he has kept the disclosure of the wonders for, we suppose, the "second edition;" and if we cannot have them just at the moment, it is well to know that they are to appear *in futuro*.

In page x Mr. Tuson discourseth in the following wise, "the brain and spinal marrow have entering into their formation *certain acids* and products which are not present in other structures, and *which are* perfectly distinct from *other animal tissues*." There are several (doubtless valuable, though to our limited comprehension somewhat obscure) scraps of information contained in this sentence. We learn, for example, that "certain acids" are "animal tissues," and yet further, that these "certain acids" are "perfectly distinct from *other* animal tissues." We have worked at this till our brain has reeled, and yet fathom its entire depth we cannot. It is indeed clear there is a *lapsus* somewhere, and we would venture to suggest that Mr. Tuson was "called to a case" at the words "certain acids," and was thereby unfortunately "*prevented from continuing the same line of argument*." That no effort of his could ever recall the profound train of thought, in which he had obviously launched, when he reached the words "certain acids," is not in the remotest degree strange. Your twelve-and-sixpenny Dutch clock may be shaken and shaken, and put out of sorts in all manner of ways, and yet on it goes still;—but your finely and delicately organized repeater must be more gently treated,—misuse it, and its train of action cannot be coaxed back into regularity. Need we say that Mr. Tuson's mind is among minds what the most precious of repeaters is among time-pieces?

Mr. Tuson taketh high rank among organic chemists: "*I*," he says, "have pointed out that brain and nervous matter contain cerebric acid, oleophosphoric acid, cholesterine, &c." We certainly never knew till this moment that it was Mr. Tuson who "pointed out" these facts; but it is never too late to learn. "What effect," he asks, "would cerebric acid have in certain deranged states of the mind? What effect oleophosphoric acid?" Aye indeed, what effect? These are certainly puzzlers for our weak noddles; but no wonder, for even our philosopher himself confesses they are questions "he is *at present* unable to answer." We do not despair, however; "in a short time he hopes to be able to place before the public some interesting particulars on this and other points." May time flow rapidly till the blissful advent of the said particulars!

Mr. Tuson next saith: "Liebig has pointed out that certain *chemical changes* occur in the lungs, differing from *other parts of the body*." Liebig has unquestionably done and said strange things, but if he has spoken of "chemical *changes*" which "differ from other *parts* of the body," then hath he outsaid himself. Our active friend was evidently called out here to another case. But if Liebig outsays himself, Mr. Tuson does more, he even outdoes himself,—only fancy, he "*looks forward* to an advancement in the scientific practice of our profession, to an extent of improvement that *cannot be here anticipated*." He looks forward to that which cannot even be anticipated! How he manages this, we are left to divine, as he immediately turns to a just denunciation of those "*members* who fancy themselves *branches* of a liberal profession, who on many occasions have been pleased to term my endeavours to advance the practice of medicine 'Quackery.'" Who are these audacious and outrageous "members" who go the length not only of "fancying themselves branches," but even of fancying Mr. Tuson a Quack? Give us their names—that we may hold them up to deserved execration.

Mr. Tuson proceedeth again to discourse chemically: proteine becomes the subject of remark. "Scherer analysed this *original matter* prepared from animal albumen and fibrine, from the crystalline lens, from hair and horn, and the results of all these analyses agreed with the formula $C_{48}\ H_{36}\ N_{6}\ O_{14}$, *which is about identical with the blood in a healthy state*." This remarkable statement of the almost identity of a chemical formula and healthy blood ushers in another no less striking. "From a variety of practical observations, made during a period of nearly twenty-five years, I have been frequently struck with the want of power in the system to enable the constitution to bear up against disease." Profound and startling discovery! After twenty-five years of labour (active labour, too, as we have already said) Mr. Tuson has succeeded in establishing to his satisfaction the novel and momentous truth that people may die of disease!

> Quis potis est dignum pollenti pectore carmen
> Condere, pro rerum majestate hisque repertis?
> Quisve valet verbis tantum, qui fundere laudes
> Pro meritis Ejus possit, qui talia nobis
> Pectore parta suo, quæsitaque præmia liquit?

The arrangement of his book naturally occupies Mr. Tuson's attention in the preface, and among his comments thereon we find the following: "Next follows the classification of diseases of the breast, *which* have been divided into three." What have been divided into three? not the classification surely,—nor the diseases, for the learned author admits some forty or so;—nor the breast, for breast is a singular noun, and Mr. Tuson has too deep a respect for Lindley Murray to give it a plural verb. What then *has* been divided into three? of this we fear we must be content to remain in ignorance; while we attend Mr. Tuson to the following pages, where he complacently refers to "*many interesting practical remarks, of service to those afflicted with abnormal formations,* contained in certain parts of his volume;—also to "considerations . . . . which terminates;"—also, "to the following works," which following works, be it observed are *names of writers* simply, one half of the foreign ones being mispelled, &c.

"Finally," says our modest author, "all reasonable means have been bestowed to render my labour complete, yet after all there may be certain parts which require correction and improvement." Let no such misgivings assail thee, O great man,

> E tenebris tantis tam clarum extollere lumen
> Qui primus potuisti:—

nothing can be more complete or perfect *in its way* than thy volume,—to alter would be but to spoil;—to improve would be impossible.

But to descend from the heights of Tuson to the level of common men and common physic:—it does not require the experience, which our habit of examining books critically has of necessity given us, to know that the author of such a preface, as that just noticed, must be incapable, under any estimate of human possibility, of writing a treatise worth reading upon any possible subject. But it may be that the reader desires some proofs of the fact, as deducible from the volume before us; and though we confess ourselves most strongly disinclined to the task, we proceed to discharge, as quickly as possible, what perhaps may be esteemed a duty. But of analysing the book we have no intention,—indeed it defies analysis,—we shall simply give *a few instances,* as they chance to fall under our eye, of the various and manifold *peculiarities* which pervade the pages before us, and which, though we mortal critics may deem them blots, or blunders, or blemishes, may, in reality, be in the eyes of the gods and Mr. Tuson, beauties of the first water:—to enumerate all would be tantamount to reprinting the work—minus some of its quotations.

Mr. Tuson discourseth concerning the importance of *classification,* and exhibits his power in this way by placing "hysterical affections of the breast" among the "Organic Lesions" of that part. (p. 134.) But this is a small effort: see the classification of "cancerous diseases." (p. 219.)

| | |
|---|---|
| **Scirrhus.** | **Carcinoma simplex.** |
| **Cancer, fungus hæmatodes, medullary sarcoma, and melanosis** | **Carcinoma reticulare**<br>**" alveolare**<br>**" medullare**<br>**" melanodes**<br>**" fasciculatum.** |

Was there ever such an attempt made at classification since the creation of scatter-brains? Each word absolutely constitutes or implies an error—that is, to us, critics of the earth, earthy: but, Dîs et Tusoni aliter visum.

Mr. Tuson occasionally talketh concerning subjects of which (with all his "activity") he may be presumed not to be over deeply acquainted.

He dippeth deeply into the fantastic chemistry of the day,—but in a mighty safe way, by quoting *monster-passages* from Liebig, of the real signification of which he probably comprehends no more than of so many problems in astronomy. Now we do not expect Mr. Tuson to be a chemist; he has enough to do—or should have enough to do—without wandering from the regions of practice; but we do expect a great pathologist like him—one leading on the front ranks of "active" pathological investigation—to have at least some glimmering of accuracy in his ideas on such subjects as the generation of pus. Yet truth—for which our veneration is greater than even for Tuson—compels us to affirm that the man's mind is in a state of Cimmerian darkness on the subject. Conceive him—at the present hour—quietly accepting the notion of pus-globules being produced (in ordinary suppuration) in the interior of the vessels from changes of the red corpuscles of the blood, and admitting by inference the heresy in physiology that the capillaries have open mouths!

Mr. Tuson's singular power of appreciating the bearings of a question of morbid anatomy, and of his grand off-hand way of getting rid of small difficulties that trouble weaker minds is deliciously exhibited in his account of "fibrous tumours of the breast." This account is simply a *reprint* from an English journal of M. Cruveilhier's opinions (as these existed previous to the discussion at the Paris Academy) on the subject; it occupies *eighteen* pages of our learned author's volume, and is put down without one syllable of comment in any form or shape. Now, as is notorious (we should have imagined that even Mr. Tuson knew this), Cruveilhier was obliged, during the progress of discussion, to retract many of the positions which he started with; what is printed by Mr. Tuson consequently misstates the existing opinions of Cruveilhier; and gives aid (such as it is) in disseminating abundant error. What singular obliquity of intellect—that betrays the author, even in reprinting huge masses of other people's writings, into flagrant perversion of historic and actual truth!

One method by which Mr. Tuson evidently fancies he produces a very striking effect, is by suddenly turning off from the subject before him to some other utterly unconnected topic: the specimens of such "Tusonian mental catenation," as we would venture to christen it, are really wonderful. Chemistry is the subject to which the wandering intellect constantly turns; and may be termed the ignis fatuus of the Tusonian mind. Thus, among a multitude of such vagaries, Mr. Tuson in one place discourses concerning the process of suckling and the properties of human milk, and then and there launches into *quotations* of *eleven* pages in length from Turner and Liebig, concerning the composition of the animal textures. These quotations are a tolerably safe game (not always so, however, as appears from the instance already given); but the moment Mr. Tuson becomes chemical on his own resources an occasional Tusonianism of course occurs. Thus appears the phrase, "caseine which is distinguished from fibrine and albumen by not coagulating when heated;" (p. 23)—"coagulation by heat" is somewhat of a novel characteristic of fibrine, we believe.

The chapter on cancer displays another of the peculiarities of our author. All manner of opinions are quoted with approbation; they are all of them right; and yet these opinions are, in numerous instances, more or less totally subversive of each other. In the very narrative of these opinions there is curious disorder; it would seem as if the facetious Tuson had put his quotations on separate pieces of paper, shaken them in a bag and then printed away on the principle of first come first served. But there is much novel instruction in some parts of this chapter; such as that derivable from the fact that Mr. Tuson describes "fungus hæmatodes," (p. 315,) "carcinoma medullare," (p. 331,) and "medullary sarcoma," (p. 343,) as though they were perfectly different productions!

In describing atrophy of the breast Mr. Tuson misses the only point of interest connected with that state,—namely, the possibility (more than once actually realized) of its being mistaken for the form of scirrhus attended with diminution of size of the mammary gland. The chapter on hypertrophy, on the other hand, exhibits the author's total ignorance of the only valuable essays ever produced on the subject,—among others of that of Fingerhuth.

We cannot refrain from copying some few sentences wherefrom further idea may be formed of the lucidity and perspicuity of the Tusonian style; —and we give them as they stand, without comment or illustration, however temptingly they solicit this:—"If the mother cannot, when her child by the most pathetic cries demands, yield it a genial balmy food, uninjured by fatigue, agitation of mind or indigestion." (p. 22.)—"There are certain constitutions unfit and improper to the performance of lactation."—"The intermarriages of cousins have been fully established to produce weak and delicate children, *by the admixture of the blood of one branch.*" (p. 61.)—"The deaths in Paris and its immediate environs (within a radius of five to six miles) from cancer in 1830, 668 persons were said to have died of cancerous complaints, which was 1—96 per cent. of the deaths in that year." (p. 252.)—"The maternal office of suckling is always attended with a calm serenity of mind, scarcely felt *in other situations.*" (p. 40.)—"It is stated upon authority that girls *of the best character*, by the irritation of a child sucking, have become able to support it."

Now against all these innumerable forms and varieties of defect—or to speak safely, peculiarity—what counterbalancing merits are to be placed in the scales? We shall not say—not a single one; but truly not a single one that we, mortal men, have been able to discover! We beg pardon—there is one—the greater for its very loneliness—namely, the modest delicacy of expression that pervades these pages, especially visible when Mr. Tuson has to speak of certain unmentionable parts of the frame. Thus Mr. Tuson would not for worlds talk of putting leeches to the pudendum, anus, or perinæum— he would "apply ten leeches *so as to abstract blood from the portal system.*" He writeth for mothers of families, and would not offend their chaste eyes or ears,—he looketh forward to a domestic edition for their especial use, and desireth to save his editor the task of "expurgating,"—indeed he evidently liketh not the idea of a "Tuson Expurgatus." How the mothers of families are to guess where the leeches may best be made to act on the "portal system" does not clearly appear,—but Mr. Tuson is doubtless always to be found, and always ready to listen to the cries and suffering of ignorant humanity.

It is with feelings of severe pain and annoyance that we find this book dedicated to that truly excellent man, Lord Northampton, in his capacity of President of the Royal Society. It is true that Mr. Tuson is (by what singular coincidence of chances remains a curious problem for solution) a Fellow of that Society; and it may be held a matter of common courtesy on the part of the President to accept a dedication offered by any of the Fellows. We doubt not that this was the case in the present instance. The noble Lord was assuredly, unaware, when he granted his permission, that he was about to lend the influence of his scientific position to furthering the objects of a man who is not only ignorant of science, but actually incapable of writing his mother-tongue with the degree of correctness which, now-a-days, may reasonably be expected from a maid-servant. Mr. Tuson assures us that though he has been accused of quackery he is not a quack. We take his word for the truth of what he should know better than anybody else. But if Mr. Tuson really were a quack, we should expect to see him employing the presidential name, thus incautiously accorded, precisely on the same principle as "Professor Holloway" heads his placards with the respectable and respected name of the senior Physician of Guy's Hospital.

---

